# Order through Disorder: Hyper-Mobile C-Terminal Residues Stabilize the Folded State of a Helical Peptide. A Molecular Dynamics Study

**DOI:** 10.1371/journal.pone.0015290

**Published:** 2010-12-20

**Authors:** Kalliopi K. Patapati, Nicholas M. Glykos

**Affiliations:** Department of Molecular Biology and Genetics, Democritus University of Thrace, Alexandroupolis, Greece; National Institute for Medical Research, Medical Research Council, United Kingdom

## Abstract

Conventional wisdom has it that the presence of disordered regions in the three-dimensional structures of polypeptides not only does not contribute significantly to the thermodynamic stability of their folded state, but, on the contrary, that the presence of disorder leads to a decrease of the corresponding proteins' stability. We have performed extensive 3.4 µs long folding simulations (in explicit solvent and with full electrostatics) of an undecamer peptide of experimentally known helical structure, both with and without its disordered (four residue long) C-terminal tail. Our simulations clearly indicate that the presence of the apparently disordered (in structural terms) C-terminal tail, increases the thermodynamic stability of the peptide's folded (helical) state. These results show that at least for the case of relatively short peptides, the interplay between thermodynamic stability and the apparent structural stability can be rather subtle, with even disordered regions contributing significantly to the stability of the folded state. Our results have clear implications for the understanding of peptide energetics and the design of foldable peptides.

## Introduction

**Figure 1:**
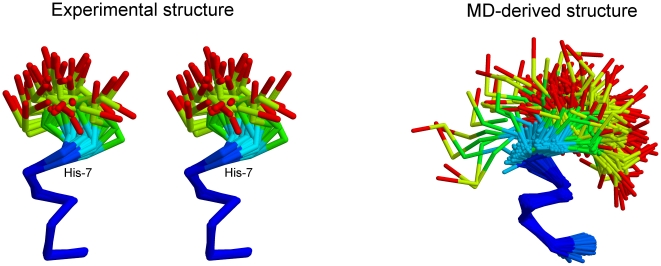
Experimentally-determined vs. molecular dynamics-derived structures. The panel on the left is a wall-eyed stereodiagram of the solution structure of the peptide (PDB entry 1CB3, all thirty deposited models are shown superimposed, structure determined at 283 K). The right panel shows a superposition of 920 structures (recorded from the molecular dynamics trajectory, simulation performed at 320 K) that belong to the major conformational cluster. For both panels, only the C_α_ traces are shown, and the bonds are colored accordingly to the root mean squared fluctuations of the participating atoms from blue (low rmsf) to red (high rmsf). The molecular dynamics-derived structures have been superimposed using the C_α_ atoms of residues 2–6 (inclusive).

Understanding peptide folding has attracted significant attention in recent years [1]–[4], with published results covering the whole range from experimental [5]–[8] to theoretical and computational approaches [9]–[12]. The recent advances in simulation algorithms (especially PME-based full electrostatics [13] and multiple time-stepping methods [14]) together with the ever-increasing availability of computing power, has made possible the application of high quality (explicit solvent, full electrostatics) simulations in the µs regime and higher. At least for short peptides, such long simulation times reduce the interpretation ambiguities associated with insufficient sampling, and are being seen as an acid test for the ability of current generation forcefields to reproduce the experimentally accessible physical reality. We have been studying the application of molecular dynamics simulations to the prediction of peptide structure, with emphasis on the ability of current generation forcefields to correctly identify foldable peptides and to predict their solution structure and dynamics. An 11-mer peptide corresponding to residues 101-111 of human α-lactalbumin [15] (hereafter referred to as αLa_101-111_) drew our attention for two reasons. The first is that the peptide not only has a stable three-dimensional structure in solution (see stereodiagram of Fig. 1), but it also contains a rather rare (at least for isolated peptides) stretch of a 3_10_ helix, as evidenced by two independent NMR structure determinations [16], [17] of two closely related forms of the peptide's sequence. The presence of a rare and persistent secondary structure element was seen as an opportunity to examine whether current generation biomolecular forcefields can reproduce statistically unusual peptide conformations. The second reason that made this peptide intriguing was the rather striking order-disorder pattern observed in the experimentally determined structures: Both NMR determinations [16], [17] show the presence of a rather stable helical structure for residues 3–6 (sequence YWLA), followed by an apparently disordered C-terminal tail for the last four residues (Fig. 1). We considered this as an opportunity to not only test the ability of the forcefield to correctly identify the stably folded parts of a structure, but also to verify (or otherwise) the notion that structurally disordered parts of polypeptides do not contribute significantly to the thermodynamic stability of their folded state [18]–[20].

To tackle these questions, we performed three µs-long molecular dynamics simulations in explicit solvent and with full electrostatics of three forms of the αLa_101-111_ peptide. The first is the full length 11-mer (sequence INYWLAHAKAG, hereafter referred to as *native*, see left panel of Fig. 1) which was simulated for a total of 1.3 µs. Analysis of the native simulation would allow us to answer the first two questions, namely (a) whether a popular current generation biomolecular forcefield can reproduce a rare secondary structure element in the context of a free peptide, and, (b) whether the order-disorder pattern seen in the experimental structures is also computationally accessible. The second simulation performed corresponds to a truncated form of the peptide (sequence INYWLAH, hereafter referred to as *truncated*) which is missing the apparently disordered C-terminal tail of the native peptide, and which was simulated for a total of 1.0 µs. Finally, a 1.1 µs long simulation of mildly truncated nonameric form of the peptide (sequence INYWLAHAK, hereafter referred to as *nonamer peptide*) was also performed to characterize the dependence of our results on the length of the deletion. Comparison of the results obtained from these simulations would allow tackle our final question, that is, whether the presence of a disordered C-terminal tail has any significant effect on the stability of the folded part of a peptide. In the sections that follow, we present results from the analysis of these three simulations and discuss their implications in the context of the relationships between order-disorder and thermodynamic stability.

## Methods

### 2.1 System preparation

For all three folding simulations the starting structures (corresponding to the sequences INYWLAHAKAG, INYWLAHAK and INYWLAH) were in the fully extended state as obtained from the program Ribosome (http://www.roselab.jhu.edu/~raj/Manuals/ribosome.html). Missing hydrogen atoms were built with the program PSFGEN [21] through its VMD plugin [22] and assuming an acidic pH (with the histidine residues fully protonated). The choice to use an acidic pH was based on the published conditions reported [16], [17] for both of the experimentally determined structures of the full length peptide (PDB entries 2DX2 and 1CB3). The peptide termini were unprotected in agreement with the experimental conditions used with the 2DX2 structure. Explicit solvent orthogonal periodic boundary systems were setup using VMD and ions were added to a final equivalent concentration of 100 mM in order to neutralize the total charge of the systems. For the truncated peptide the same system was used throughout the simulation with initial cell dimensions of 40×40×40 Å^3^, an initial shortest solute-solute distance of 14 Å, and a total of 5897 atoms of which 128 peptide atoms, 5766 water atoms (corresponding to 1922 TIP3 molecules), 1 sodium and 2 chloride ions. For the full length peptide simulation, and as the peptide assumed its persistent compact (helical) conformation, two cycles of reduction of the number of waters were undertaken. The initial system had cell dimensions of 60×60×60 Å^3^, an initial shortest solute-solute distance of 20 Å, and a total of 20475 atoms of which 177 peptide atoms, 20286 water atoms (corresponding to 6762 TIP3 molecules), 5 sodium and 7 chloride ions. Following the two cycles of water reduction, the final system had cell dimensions of 40×40×40 Å^3^, an initial shortest solute-solute distance of 22 Å, and a total of 6352 atoms of which 177 peptide atoms, 6171 water atoms (corresponding to 2057 TIP3 molecules), 1 sodium and 3 chloride ions. Finally, for the nonamer peptide the system had cell dimensions of 40×40×40 Å^3^, an initial shortest solute-solute distance of 12 Å, and a total of 5882 atoms of which 160 peptide atoms, 5718 water atoms (corresponding to 1906 TIP3 molecules), 1 sodium and 3 chloride ions.

In addition to these three folding simulations, we have additionally performed a 0.4 µs long simulation of the 11mer (full-length) peptide but starting from the experimentally determined structure and at the lower temperature of 283 K (matching the reported temperature for the experimental work). That system had cell dimensions of 40×40×40 Å^3^, an initial shortest solute-solute distance of 14 Å, and a total of 5890 atoms of which 177 peptide atoms, 5709 water atoms (corresponding to 1903 TIP3 molecules), 1 sodium and 3 chloride ions.

The topology and parameter files used throughout the system preparation were those of the CHARMM22 force field [23] (version c36a2) with the CMAP correction [24] (hereafter referred to as the ‘CHARMM forcefield’).

### 2.2 Simulation protocol

We followed the dynamics of the native (full length) peptide for a total of 1.3 µs using the program NAMD [21] and the CHARMM forcefield with the CMAP correction [24] as follows: The system was first energy minimized for 1000 conjugate gradient steps followed by a slow heating-up phase to a final temperature of 320 K (with a temperature step of 20 K) over a period of 32 ps. Subsequently the system was equilibrated for 10 ps under NpT conditions without any restraints, until the volume equilibrated. This was followed by the production NpT run with the temperature and pressure controlled using the Nosè-Hoover Langevin dynamics and Langevin piston barostat control methods as implemented by the NAMD program (and maintained at 320 K and 1 atm). The Langevin damping coefficient was set to 1 ps^−1^, and the piston's oscillation period to 200 fs, with a decay time of 100 fs. The production run was performed with the impulse Verlet-I multiple timestep integration algorithm as implemented by NAMD. The inner timestep was 2 fs, short-range non-bonded interactions were calculated every one step, and long-range electrostatics interactions every two timesteps using the particle mesh Ewald method with a grid spacing of approximately 1 Å and a tolerance of 10^−6^. A cutoff for the van der Waals interactions was applied at 8 Å through a switching function, and SHAKE (with a tolerance of 10^−8^) was used to restrain all bonds involving hydrogen atoms. Trajectories were obtained by saving the atomic coordinates of the whole system every 0.8 ps.

The truncated and nonameric forms of the peptide were simulated using exactly the same protocol as described above, for a total of 1.0 and 1.1 µs respectively.

Finally, for the 0.4 µs long simulation of the full-length peptide which was started from the experimentally-determined structure (2DX2, model 1) we also used the same protocol, with the exception that the target temperature was set to 283 K.

### 2.3 Trajectory analysis

**Figure 2:**
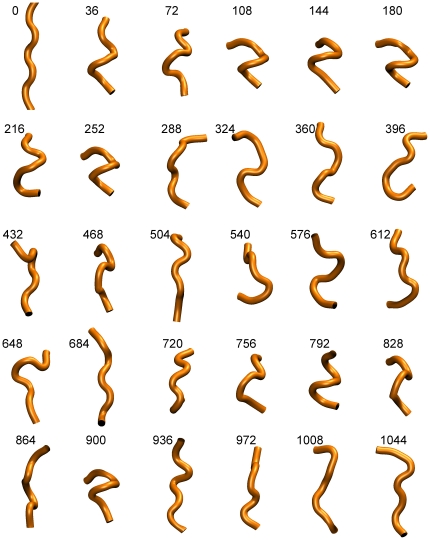
Snapshots from the trajectory of the truncated peptide. Equally spaced in time snapshots from the truncated peptide simulation. These are cartoon representations of the peptide's backbone conformation. The timings shown are in nanoseconds. The C_α_ atoms of residues 2–6 (inclusive) have been used to remove overall rotations/translations and to align the structures shown.

The program CARMA [25] was used for most of the analyses, including removal of overall rotations/translations, calculation of RMSDs from a chosen reference structure, calculation of the radius of gyration, calculation of the average structure (and of the atomic root mean squared fluctuations), production of PDB files from the trajectory, Cartesian space principal component analysis [26], [27] and corresponding cluster analysis, dihedral space principal component analysis [28], [29] and cluster analysis, calculation of the frame-to-frame RMSD matrices, etc. Secondary structure assignments were performed with the program STRIDE [30]. All molecular graphics work and preparation of Fig. 2 were performed with the program VMD [22].

### 2.4 Sufficient sampling

We calculated the eigenspace overlap between the eigenvectors obtained by dividing the native, truncated and nonamer trajectories into two non-overlapping halves (ignoring the first 80ns of each simulation) and performing PCA on each half. The Cartesian PCA-derived overlap using the top three eigenvectors is 0.98 for the native simulation, 0.93 for the truncated peptide and 0.92 for the nonamer peptide. The dihedral PCA spaces –and in agreement with previous studies [28], [29]– converge more slowly with corresponding values of 0.94, 0.67 and 0.70 using the top six eigenvectors for the native, truncated and nonamer peptides respectively. See Supporting Information S1 for the corresponding diagrams.

### 2.5 Selection of representative helical structure

The representative helical structure of the native trajectory was selected as follows: over all structures belonging to the major conformational cluster, an average peptide structure was calculated (in Cartesian space and following removal of overall rotations/translations). The native trajectory's structure with the smallest rms deviation from this average structure (recorded at approximately 0.65 µs with an rmsd of approximately 0.15 Å) was selected as the simulation's native state.

### 2.6 Selection of folded structures

All structures with an rms deviation of less than 0.8 Å (from the representative, see previous section) were taken to be in the ‘folded’ state, and all structures with an rmsd higher than 0.8 Å to be in the ‘unfolded’ state. The cutoff of 0.8 Å was determined as follows: a histogram of the rmsd's from the representative helical structure was prepared for the truncated peptide using a step 0.05 Å (see Supporting Information S1). In this histogram the folded (helical) structures gave a clear peak (corresponding to low rmsd's) separated by the next peak (at higher rmsd's) at a midpoint of 0.8 Å. The thus determined cutoff was subsequently applied to both the native and truncated peptide simulations.

### 2.7 Computational means and requirements

All simulations were performed on a stateless Beowulf cluster (http://norma.mbg.duth.gr) based on quad core processors on a gigabit interconnect using an SMP-enabled version of NAMD v.2.7b. Due to the small size of the systems, the simulations only scaled to a total of eight cores, giving a maximum observed throughput of ∼21 ns/day for a 6,000 atom system, and requiring for all reported simulations the equivalent of a grant total of approximately 180 days of physical time on eight dedicated cores.

## Results

### 3.1 The full-length peptide folds quickly and is surprisingly stable

**Figure 3:**
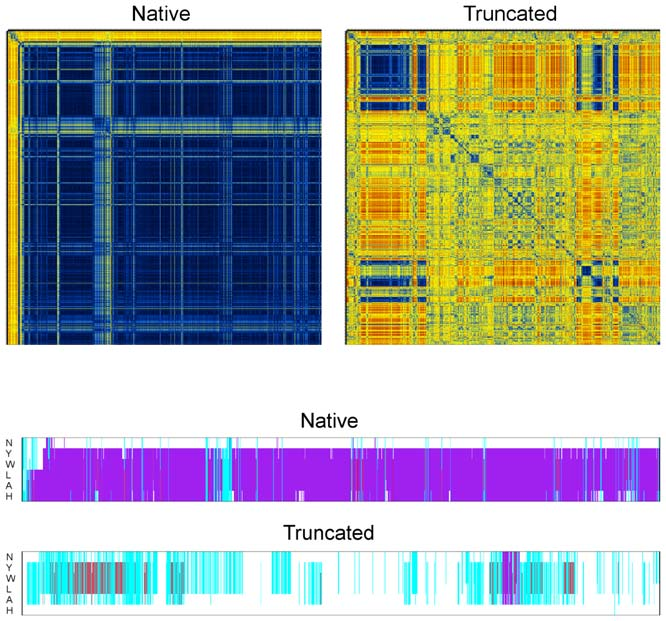
RMSD matrices and secondary structure assignments. The two images on the upper panel depict using a color representation the values of the root mean squared deviation between all possible pairs of structures recorded from each of the two trajectories. The origin (corresponding to *t = 0*) is at the top left-hand corner of each matrix, with successive structures corresponding to successive image points both along the horizontal and the vertical axes. Both matrices were calculated using the C_α_ coordinates of residues 1–7 (inclusive). The two images are on the same color scale, ranging from dark blue (corresponding to an RMSD of zero), through yellow (for RMSDs of about 2.6 Å), to dark red (corresponding to an RMSD of 5.3 Å). The lower panel shows the STRIDE-derived secondary structure assignments per residue vs. time for the indicated simulations. Assignments for residues 2–7 (inclusive) are shown with the states being α-helix (mauve), 3_10_-helix (red), turn regions (cyan), coil (white).

**Figure 4:**
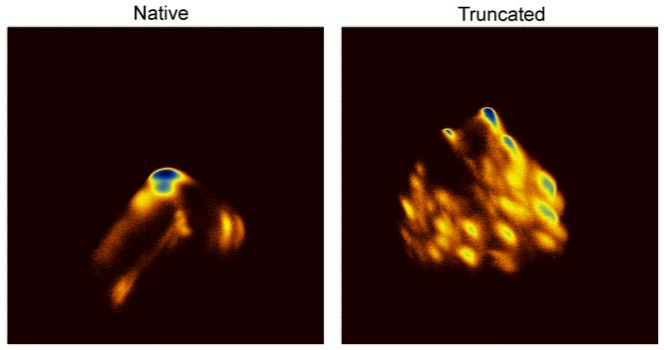
Dihedral-angle principal component analysis. The two images depict using a color representation the density maps corresponding to the projection of each trajectory on the plane defined by the first two principal components (higher density is blue). Because the logarithm has been taken, these images can be viewed as free energy landscapes (using as order parameters the two major principal components), with corresponding minima of −4.4 kcal/mol for the native trajectory and −3.8 kcal/mol for the truncated peptide. For both diagrams, twelve ϕ,ψ torsion angles obtained from the first seven residues were used for the analysis. The images are on the same scale (ranging from −4.5 to +4.5 on both axes), the origin is on the top-left-hand-side corner, and the first principal component is plotted on the vertical axis.

**Figure 5:**
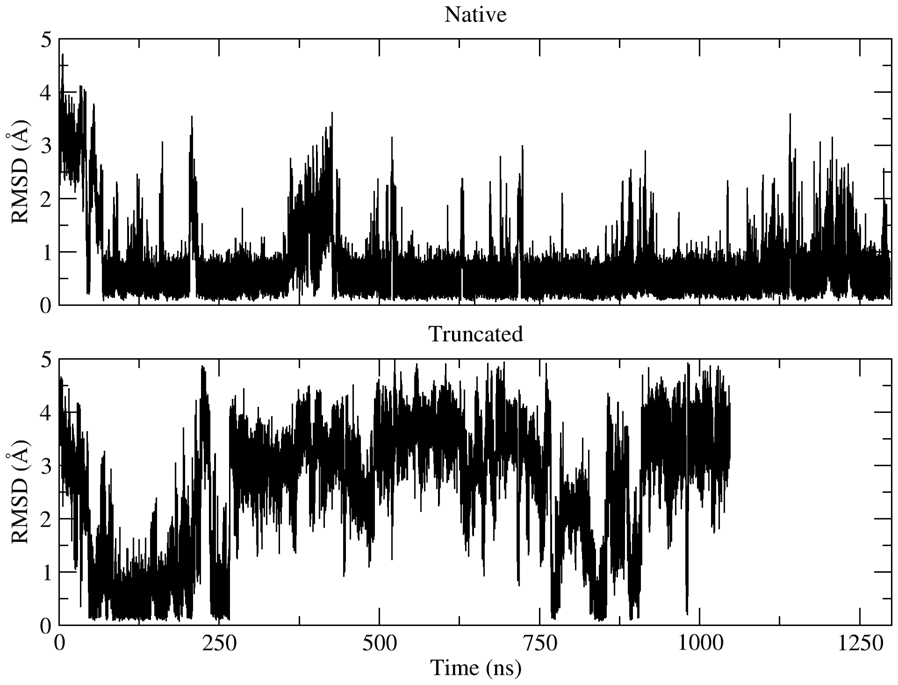
Similarity with the folded (helical) structure. These graphs show the variation (versus simulation time) of the RMSD between each of the two trajectories' structures, and the folded simulation-derived (α-helical) structure. For the calculation we used the C_α_ coordinates of residues 1–7 (inclusive). The folded (helical) structure was taken to be the structure recorded from the native trajectory which had the lowest RMSD from the average.

Figure 1 (right panel) shows a superposition of 920 structures obtained directly from the native trajectory and representing well over 80% of the trajectory's total length (corresponding to the persistence time of the major conformational cluster, see below). The molecular dynamics-derived structure, although obtained at the significantly higher temperature of 320 K, shows pronounced similarities with the experimentally determined structure (obtained at 283 K, Fig. 1): Both structures show a characteristic bipartite organization with a very stably folded helical N-terminal part (colored blue in Fig. 1), and a highly mobile C-terminal tail. To put this observation in numbers, we calculated the average atomic root mean squared fluctuations for two disjoined sets of atoms: For the C_α_ atoms of residues 1–7 (inclusive) we obtained an average atomic rms fluctuation of only 0.4 Å, an order of magnitude smaller than that of residues 8–11, standing at a hefty 4.1 Å. The stability of the folded (N-terminal) part of the peptide is easier to visualize by examining the rms deviations between all possible pairs of structures obtained from the native trajectory, as shown in the rmsd matrix of the upper panel of Fig. 3: The initial extended (unfolded) structure within only 70 ns converges (for its N-terminal part) to a stable structure which persists almost continuously for the remaining 1.2 µs of the simulation [the transition just described corresponds, in Fig. 3, to the passage from the initial yellow-red stripe (high values of rms deviation), to the almost wholly dark blue part of the matrix (low rmsd values)]. Principal component analysis in dihedral (ϕ,ψ) space supports the same conclusions: As shown in the left panel of Fig. 4, the projection of the native trajectory on the plane defined by the first two principal components is dominated by a single slightly bilobal peak accounting for more than 80% of the total simulation time and corresponding to the helical structure of residues 1–7 (as shown in Fig. 1). The other populated areas of the diagram correspond to the structures visited by the peptide during the major folding event (0–70 ns of the simulation), as well as two partial unfolding-refolding events recorded at about 375 and 1200 ns respectively. These transitions are easier to visualize through the calculation of an order parameter which is directly related to the folded structure of the peptide, as shown in Fig. 5. The upper diagram of this Figure shows the evolution (versus simulation time) of the rms deviation between each structure recorded from the native trajectory and a representative structure (see section 2.5) corresponding to the peptide's folded state [only the C_α_ atoms of residues 1–7 (inclusive) were used for this calculation]. As can be seen from this diagram, and in agreement with the preceding analyses, the native peptide quickly folds (for its N-terminal part) to its native state and remains there for almost the whole length of the trajectory. The partial unfolding events mentioned earlier, can now easily be identified as high rmsd areas centered at about 375 and 1200 ns respectively. Based on this Figure, we can obtain a very rough estimate of the simulation-derived free energy of folding of the native peptide at 320 K as follows: Taking all structures with an rms deviation of less than 0.8 Å (from the representative) to be in the ‘folded’ state (see section 2.6), and all structures with an rmsd higher than 0.8 Å to be in the ‘unfolded’ state, we obtain a Δ*G_folding_*  =  –*R* T log(*p*_folded_/*p*_unfolded_)  =  –*R* T log(0.80/0.20)  = –3.7 kJ/mol at 320 K, where *p*_folded_ and *p*_unfolded_ are the simulation-derived probabilities of observing the peptide in the folded and unfolded state respectively. Such large (for a peptide) values of Δ*G_folding_* together with the apparent structural stability noted in all of the preceding analyses, deserve some discussion, especially in connection with the experimental results of Araki & Tamura [16]. These authors, obtained a value for *p*_folded_ of only 0.20 (at 0°C) based on their NMR data, and a value of 0.44 from their far UV CD spectra, corresponding to values of Δ*G_folding_* of +3.3 kJ/mol and +0.6 kJ/mol respectively. The large differences between the experimental and simulation-derived free energies of folding clearly indicate that the simulation significantly over-stabilizes the helical conformation. The simulation's helical bias is discussed more extensively in the next section.

To summarize the results from this section, analysis of the native trajectory showed that (a) the N-terminal part of the full-length peptide folds quickly to a helical structure, (b) this helical structure is exceedingly stable during the simulation, far more stable than what would be expected based on the available experimental data, and, (c) the order-disorder pattern observed in the experimental (NMR) structures is faithfully reproduced by the forcefield.

### 3.2 The simulation fails to reproduce the experimentally observed 3_10_ helix

The lower panel of Fig. 3 shows the per residue secondary structure assignments (versus simulation time) as obtained with the program STRIDE. The results are exceptionally clear and in full agreement with the superimposed structures shown in the right panel of Fig. 1: The native simulation is fully biased towards the α-helical state and fails to reproduce the experimentally observed 3_10_ helix (this can also be inferred by comparing the structures depicted in Fig. 1). To take this observation a step further, we note that this α-helical bias is not due to a failure of the simulation to visit 3_10_-like structures. Indeed, calculation of the rms deviation between the experimental 2DX2 structure and each of the trajectory's structures (using, again, only the C_α_ atoms of residues 1–7), showed that the simulation repeatedly visited 3_10_-like structures, with recorded rms deviations as low as 0.48 Å (at ∼1.2 µs) and 0.65 Å (at ∼0.38 µs). We note that the time intervals at which 3_10_-like structures were visited correspond to the short-lived unfolding-refolding events of the α-helical state (at 375 and 1200 ns) as discussed in the previous section, and shown in Fig. 5. These observations, together with the exceptional stability of the α-helical state described in the previous section, appear to unequivocally suggest that the forcefield and simulation protocol used in this study result to an energy landscape with a deep and pronounced minimum, which, nonetheless, is not the experimentally-determined one.

This last proposition is a strong one, and several objections against it can be put forward. The first has to do with the significantly different temperatures (283 vs. 320 K) used in the experiment and the simulation. To verify that the α-helical bias observed in the folding simulation is not temperature-dependant, and to re-enforce the argument made in the previous paragraph, we have performed an additional 0.4 µs-long simulation of the full-length peptide, this time at 283 K and using as a starting conformation the experimentally determined 2DX2 structure (the forcefield and simulation protocol used are the same as those described in the methods section). In that simulation, the starting structure's 3_10_ helix disappears almost immediately and is replaced with an α-helix which persists for the remaining of the simulation. The speed with which the 3_10_ helix is given-up is worth mentioning: within less than 14 ns of simulation time, all STRIDE-derived assignments are wholly α-helical, with the corresponding structures reaching an rms deviation of only 0.14 Å from the representative α-helical structure (of the folding trajectory) in less than 60 ns. These results clearly show that (a) the 3_10_-helix is not stable even at 283 K, (b) that the α-helical forcefield bias is present and strong even at this lower temperature, and, (c) that the simulations at 320 K and 283 K are fully consistent with each other with respect to the stability and structure of the peptide's folded part.

A second possible explanation for the apparent disagreement between experiment and simulation would be based on refuting the single-structure-based interpretation of the NMR data, and on evoking an argument based on averaging between distinct and fast-interconverting peptide structures. The number and presence of strong and clear ROEs connecting carbonyl oxygens and amide protons of pairs of (i,i+3) residues in both structure determinations [16], [17] leaves little doubt that this is not the case.

In summary, the forcefield used in this study –and in agreement with previous studies [31]–[33]– shows a strong α-helical bias and fails to reproduce the experimentally-determined 3_10_ helical state. It would appear that such a failure invalidates any further application of the CHARMM forcefield for studying the truncated form of the αLa_101-111_ peptide. We argue that this may not be the case, exactly because the forcefield *is* biased: A forcefield fully biased towards a folded helical state can only damp (underestimate) the destabilizing effects of mutations/deletions, especially when these mutations/deletions correspond to structurally disordered residues as judged by this same forcefield. To put this differently, if a forcefield biased towards the helical state shows that an apparently disordered tail is crucial for the preservation of the secondary structure (towards which *it is already biased*), then this would provide compelling evidence for the importance of these disordered residues in stabilizing the folded state.

### 3.3 Deletion of the disordered C-terminal tail abolishes peptide folding

Figure 2 shows a collage of structures recorded from the truncated peptide's simulation, illustrating the consequences of the deletion of the peptide's disordered C-terminal tail. With the exception of two relatively short-lived intervals centered at about 130 and 900 ns, the truncated peptide remains in an unfolded, random-coil state for almost the whole of the 1 µs-long simulation, as indicated both by the disparity and the semi-extended state of the structures depicted in Fig. 2. The pronounced consequences of the deletion on the stability of the peptide are easier to visualize by comparing the rmsd matrices shown in the upper panel of Fig. 3: whereas the native peptide maintains its helical conformation almost throughout the simulation (blue area of diagram), the truncated form spends most of its time exploring unrelated (random-coil-like) structures (yellow-red areas). The folded intervals mentioned above (∼130 and 900 ns) coincide with the blue areas of this diagram. The multitude of different structures visited by the truncated peptide during the simulation can be inferred from the right panel of Fig. 4: the projection of the truncated peptide's trajectory on the plane defined by the first two principal components contains more than twenty well-defined maxima of comparable intensity, which correspond to distinct peptide conformations. As shown in the lower panel of Fig. 3, the STRIDE-derived assignments clearly show that most of these transiently stable peptide structures are devoid of persisting secondary structure elements. The exception is, of course, the short folding events mentioned above. To characterize these we calculated, as before, the rmsd from the native trajectory's representative structure (see section 3.1). The lower panel of Fig. 5 shows that during the two folding events, even the truncated peptide assumes the native-like α-helical conformation. From this same Fig. 5, we obtain as before (using a 0.8 Å cutoff) a value for Δ*G_folding_*  =  –*R* T log(*p*_folded_/*p*_unfolded_)  =  –*R* T log(0.15/0.85)  =  +4.6 kJ/mol at 320 K, a difference (from the native peptide) of ΔΔ*G_folding_*[truncated-native]  =  +8.3 kJ/mol (at 320 K). We should note on passing, that the value obtained for ΔΔ*G_folding_* using the rms deviation from the native state as an order parameter (Fig. 5) is higher (but comparable) with the value obtained from the dihedral principal component analysis (Fig. 4) which gives a value of ΔΔ*G_folding_*[truncated-native] of +5.7 kJ/mol (at 320 K). This difference can possibly be attributed to the limited number of principal components used for the analysis (accounting for approximately 35% of the total variability).

In summary, then, deletion of the structurally disordered C-terminal tail, completely destabilizes the helical structure, converting the peptide from a very stable folder to a random-coil-like structure. The natural question that arises, is, of course, why? What is the reason (in terms of energy) that leads to such significant differences? To approach this question we went back to the native trajectory and calculated (as a function of simulation time) the non-bonded energy term between disjoined sets of peptide atoms corresponding to the N-terminal heptapeptide and residues from the disordered tail (and using a value for the dielectric constant equal to 20). The average non-bonded interaction energy between residues 1–7 (inclusive) and residue 8 was found to be –3.7 kcal/mol, between residues 1–7 and residue 9 was even higher at –4.5 kcal/mol, and for residues 1–7 with residues 10–11 (inclusive) was found to be –2.1 kcal/mol. Although these are very rough estimates, they do show the complexities arising when attempting to judge thermodynamic stability from structural rigidity. For, example, the lysine at position 9 which –with respect to the previous calculations– appears to interact the most with the N-terminal part, is highly disordered in both the experimental structure (with no ROEs associated with her), and the molecular dynamics-derived one. Having said that, comparison of the numbers quoted above with the ΔG*_folding_* values obtained for the native simulation, appears to suggest that interactions between residues 1–7 with residues Ala_8_ and Lys_9_ are crucial for the stability of the folded (N-terminal) part of the peptide.

**Figure 6:**
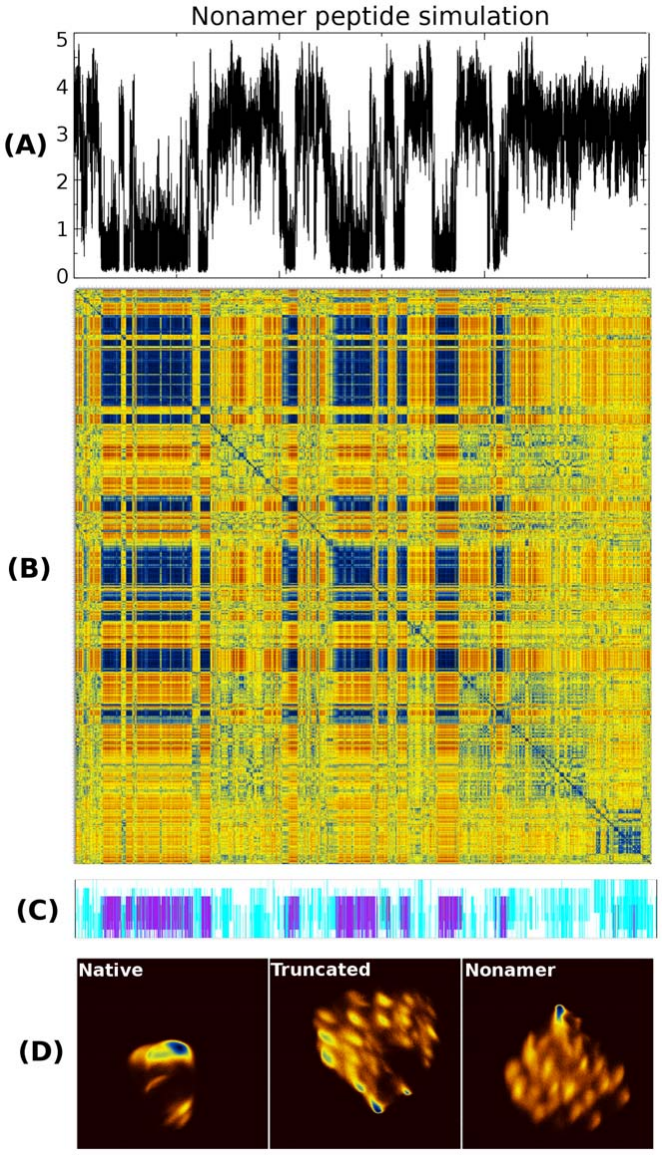
Nonamer (INYWLAHAK) peptide simulation. Panel (A) shows the variation (versus simulation time) of the RMSD between each of the trajectory's structures, and the folded (simulation-derived) α-helical structure (compare with Fig. 5). Panels (B) and (C) are the frame-to-frame RMSD matrix and the STRIDE-derived secondary structure assignments which should be compared with those shown in Fig. 3. Panel (D) shows a direct comparison between the free energy landscapes (using as order parameters the two major dPCA-derived principal components) for all three peptides studied, excluding the first 80 ns of each simulation. For the top three panels, the horizontal axes correspond to simulation time and range from 0 to 1.1 µs. Only the first seven residues have been used for all indicated analyses.

To test this hypothesis, and to establish the dependence of these results on the extend of the deletion we have performed a 1.1 µs long simulation of a form of the peptide missing only the last two residues (sequence INYWLAHAK). The results shown in Fig. 6 leave little doubt: the nonamer peptide (which includes Ala_8_ and Lys_9_) is almost as disordered (and as non-helical) as the truncated peptide, although what is missing now in terms of sequence (and with respect to the full-length peptide) is only the C-terminal alanine and glycine residues. In other words, even deletion of only the two most disordered residues, still abolishes folding of the peptide's N-terminal part. Clearly, and at least for the given peptide, estimating interaction energies using as a guide structure schematics (like those shown in Fig. 1), may lead to serious errors.

## Discussion

We have shown that, at least for relatively short peptides, even structurally disordered regions can greatly influence the thermodynamic stability of the peptide's folded state. Although such an effect is difficult to imagine taking place with large proteins, our observation does question the –usually taken for granted– idea that disordered regions do not influence significantly the stability of the structural core of a polypeptide. It could be argued, in retrospect, that there is nothing really unexpected in the results of our calculations: to use a *reductio ad absurdum* approach, who would be surprised if, say, a folded hexapeptide was shown to be destabilized by the deletion of two residues? We believe that this line of argument tacitly avoids the direct (and experimentally supported) message conveyed by Fig. 1: The real question, at least to our mind, is who would have expected that deletion of the disordered tail shown in this Figure would have such an outstanding effect on the stability of the folded part, especially when using a forcefield that we demonstrated to be heavily biased towards the folded helical state of the given system. Structural biologists tend to ignore structurally disordered regions, not the least because these regions are frequently altogether absent from the crystallographically determined structures. Our results show that ignoring disorder may hide some surprises in the case of peptides.

## Supporting Information

Supporting Information S1 **Sufficient sampling:** Eigenspace overlap as a function of the number of eigenvectors for all three simulations and using both Cartesian (left column) and dihedral (right column) PCA. **Determination of rmsd cutoff for folded structures:** Histogram of the distribution of the RMSDs of the truncated peptide's simulation from the representative α-helical structure. The folded (helical) structures correspond to the first peak from the left. The minimum of the histogram separating the folded helical structures from the next peak is at 0.78 Å (indicated by an arrow head in the figure above).
(PDF)
