# Supplementary material for: Order through Disorder: Hyper-Mobile C-Terminal Residues Stabilize the Folded State of a Helical Peptide. A Molecular Dynamics Study
Source: PLoS One. 2010 Dec 20;5(12):e15290. doi: 10.1371/journal.pone.0015290 (PMC3004869; doi:10.1371/journal.pone.0015290)
Supplement: Supporting Information S1 — Sufficient sampling: Eigenspace overlap as a function of the number of eigenvectors for all three simulations and using both Cartesian (left column) and dihedral (right column) PCA. Determination of rmsd cutoff for folded structures: Histogram of the distribution of the RMSDs of the truncated peptide's simulation from the representative α-helical structure. The folded (helical) structures correspond to the first peak from the left. The minimum of the histogram separating the folded helical structures from the next peak is at 0.78 Å (indicated by an arrow head in the figure above). (PDF) [file pone.0015290.s001.pdf]

Supporting Information

Order Through Disorder :
Hyper-mobile C-terminal Residues
Stabilize the Folded State of a Helical Peptide.

A Molecular Dynamics Study.

Kalliopi K. Patapati & Nicholas M. Glykos*

Department of Molecular Biology and Genetics, Democritus University of Thrace, University campus, 68100 Alexandroupolis, Greece, Tel +30-25510-30620, Fax +30-25510-30620, http://utopia.duth.gr/~glykos/ , glykos@mbg.duth.gr

Sufficient sampling

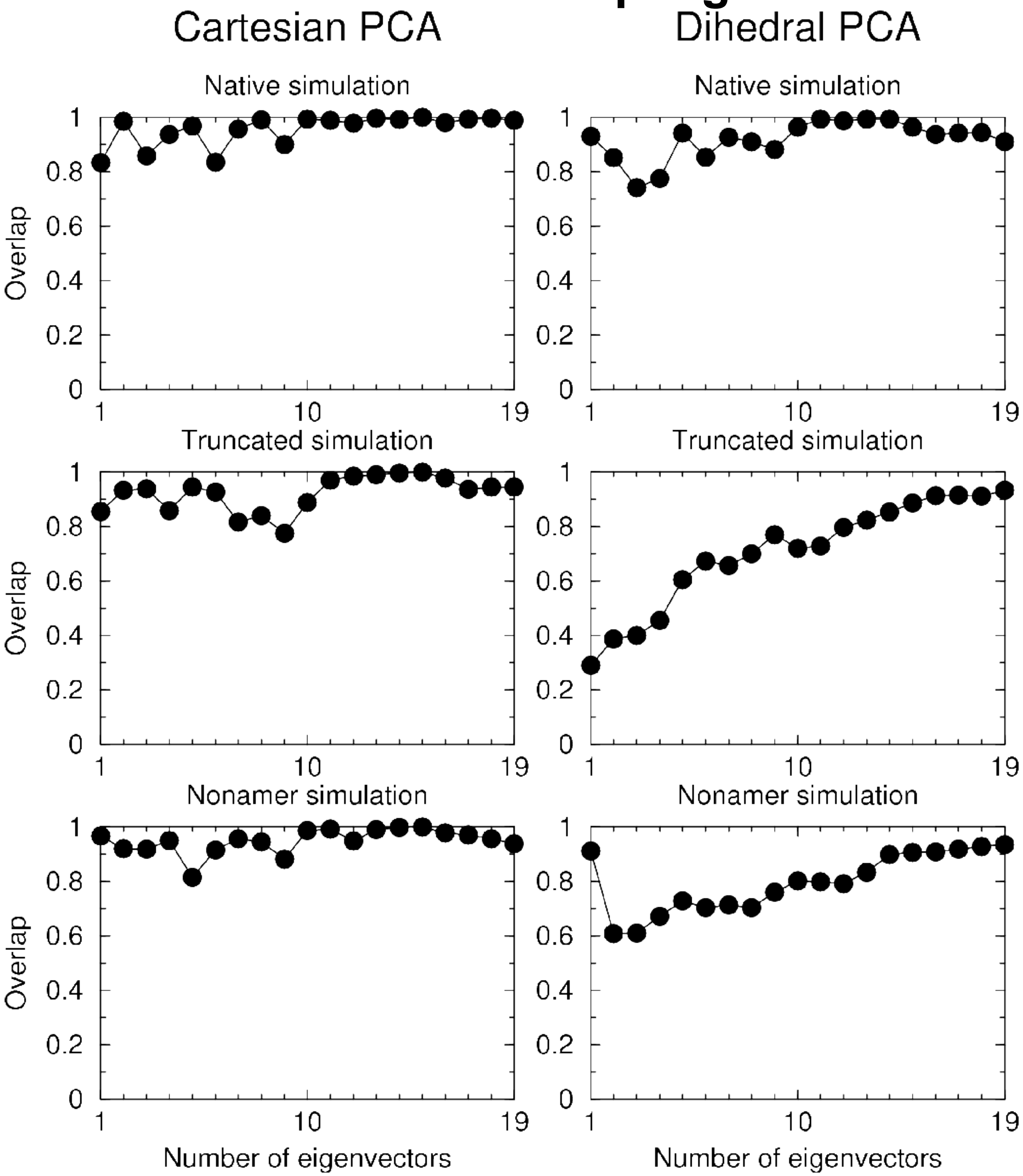

Sufficient sampling: Eigenspace overlap as a function of the number of eigenvectors for all three simulations and using both Cartesian (left column) and dihedral (right column) PCA.

Determination of rmsd cutoff for folded structures

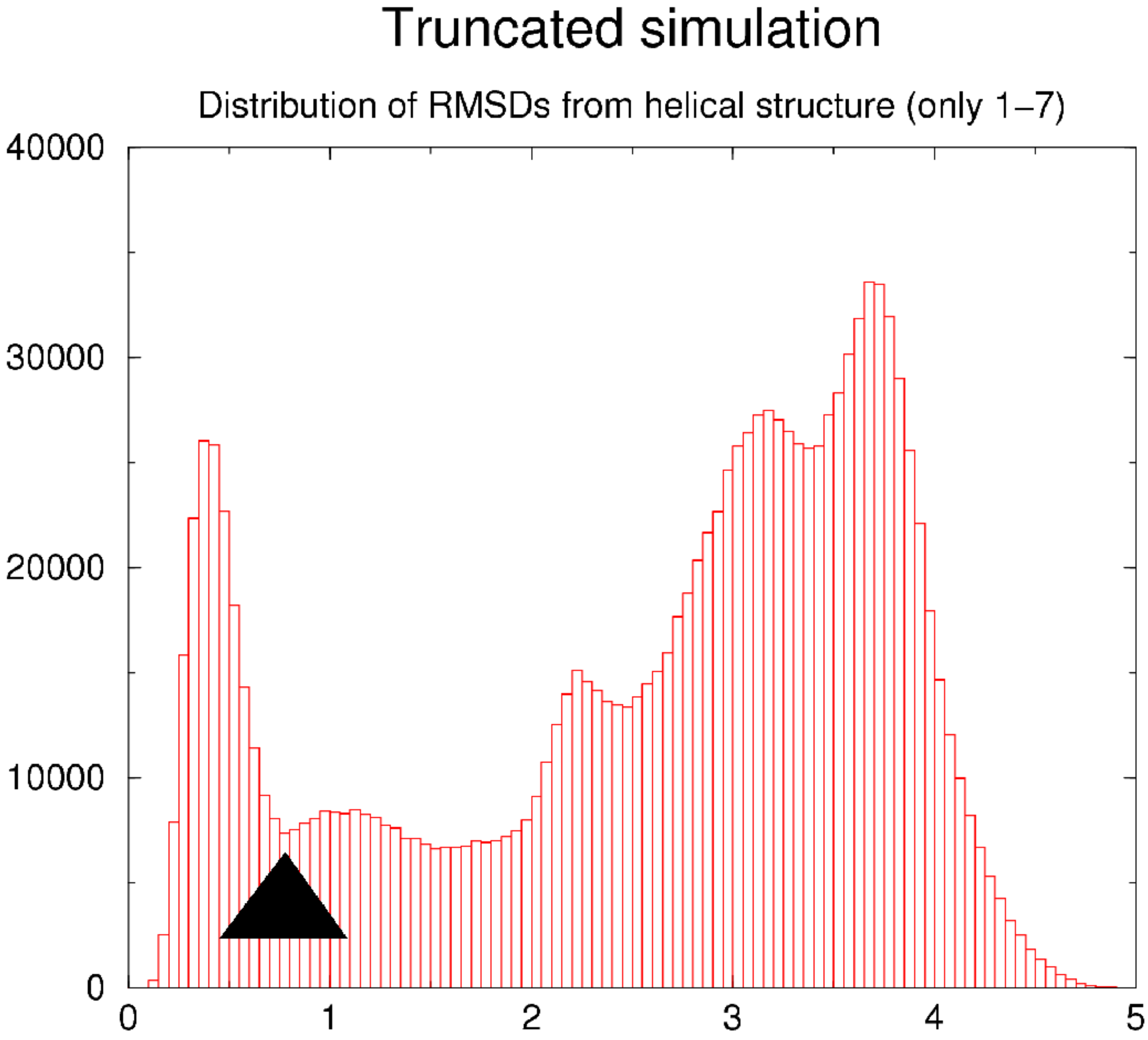

Determination of rmsd cutoff for folded structures: Histogram of the distribution of the RMSDs of the truncated peptide's simulation from the representative α-helical structure. The folded (helical) structures correspond to the first peak from the left. The minimum of the histogram separating the folded helical structures from the next peak is at 0.78Å (indicated by an arrow head in the figure above).
